# Maternal exposure to high ambient temperature and risk of stillbirth in South Australia: a statewide cohort study, 2000–2021

**DOI:** 10.1007/s00484-026-03222-4

**Published:** 2026-05-11

**Authors:** Chelsea Dyer, Adriana Milazzo, Lynne Giles

**Affiliations:** 1 School of Public Health, Adelaide University, Adelaide, South Australia Australia; 2 Robinson Research Institute, Adelaide University, Adelaide, South Australia Australia

**Keywords:** Stillbirth, Ambient temperature, Adverse birth outcomes, Climate change, Environmental epidemiology

## Abstract

Recently, pregnant women have been identified as susceptible to the effects of high ambient temperature. Evidence suggests that high temperature increases the risk of stillbirth, however, how this association differs by social disadvantage remains unclear. The aim of this study is to investigate the effect of maternal exposure to high ambient temperature on the risk of stillbirth, overall, and by social disadvantage. Maternal and birth information from 1 January 2000 to 31 December 2021 were obtained from the South Australian Perinatal Statistics Collection. We implemented a cohort study design of 417,207 births including 2,776 (0.7%) stillbirths. Mixed-effect logistic regression models with odds ratios (OR) and 95% confidence intervals (95% CI) were used to examine the effect of maternal exposure to daily maximum temperature at birth (lag day 0), and for each individual day in the week preceding delivery (lag days 1–7). Adjustments for low, medium or high disadvantage were considered in our models. Maternal exposure to high temperature ≥ 95th percentile (36.1 °C) in the week before delivery slightly increased the risk of stillbirth on lag day 2 (aOR: 1.10, 95%CI: 0.93–1.30, *p* = 0.26) and slightly decreased risk on lag day 3 (aOR: 0.90, 95%CI: 0.75–1.08, *p* = 0.26); however, the confidence intervals were wide and included 1, consistent with a null effect. Social disadvantage made negligible difference to the estimated odds of stillbirth associated with maximum temperature ≥ 95th percentile in the week before delivery. We recommend pregnant women consider adaptation strategies during periods of high temperature to minimise the risk of stillbirth.

**Supplementary Information:**

The online version contains supplementary material available at 10.1007/s00484-026-03222-4.

## Introduction

The impacts of climate change, including the increasing frequency and intensity of high ambient temperature, are one of the leading global public health threats of the 21st century (IPCC, 2023). Global temperatures have risen by 1.1 °C from 1850 to 2020 (IPCC, 2023). Australia’s climate has also warmed, on average, by 1.5 °C from 1910 to 2023, and is expected to continue to increase (CSIRO, 2024). The decade from 2011 to 2020 contains eight (2013 to 2020) of the warmest years on national record (CSIRO, 2024). South Australia (SA), the location for this study, is the driest state in the driest inhabited continent on earth (EPA, 2023) and experiences frequent high temperatures.

High ambient temperature is associated with adverse health outcomes (IPCC, 2023). Certain population groups are more susceptible to the effects of high temperature (IPCC, 2023), with pregnant women recently identified as one such group (Samuels et al. 2022). Physiological changes in pregnancy can impact thermoregulatory capabilities and include lowering of the core body temperature and sweat threshold, and increases in plasma volume, skin blood flow, and thermal heat capacity due to increased body mass (Samuels et al. 2022). These changes keep the core body temperature maintained within the normal range of 35.5–37.5 °C (Samuels et al. 2022). However, these physiological changes in pregnancy can be compromised during exposure to high ambient temperature, increasing the risk of dehydration and heat strain, especially in those with pre-existing medical conditions (Lakhoo et al. 2025; Samuels et al. 2022). Sequelae can include pre-eclampsia and gestational diabetes and adverse birth outcomes such as early pregnancy loss, congenital abnormalities, preterm birth, low birthweight, small-for-gestational age, and stillbirth (Bonell et al. 2024).

Stillbirth is a devastating outcome with multiple potential causes including environmental, social, and individual factors, the interplay of which remains poorly understood (Bowman et al. 2024; Nyadanu et al. 2022). Stillbirths may carry profound psychosocial and economic costs for affected individuals, families, and society (Haile et al. 2024). The 2014 Every Newborn Action Plan, led by WHO and UNICEF, created a global target for every country to reduce their annual stillbirth rate to 12 or fewer stillbirths per 1000 births by 2030 (GBD 2021 Global Stillbirths Collaborators 2024). Australia meets this target; however, stillbirth rates have not declined this century, remaining consistent between 6.7 and 7.7 stillbirths per 1000 births annually (AIHW, 2024). Similar patterns have been observed in other high-income countries, where stillbirth remains a serious public health issue (Flenady et al. 2016).

Recent epidemiological studies have reported an increased risk of stillbirth with maternal exposure to high ambient temperature throughout pregnancy (Auger et al. 2017; Kanner et al. 2020; Li et al. 2018; Nyadanu et al. 2022; Richards et al. 2022; Wang et al. 2019; Yang et al. 2022). Studies largely focused on the short-term effects of high ambient temperature exposure in the days or weeks prior to delivery (Wang et al. 2019). The studies differed by population size and characteristics, geographical location (most studies conducted in high-income countries), seasonal variation, climate (tropical, sub-tropical and temperate) and by temperature metrics (daily mean/maximum temperature exposure, cumulative exposure, different time lags considered) (Auger et al. 2017; Chersich et al. 2020; Kanner et al. 2020; Li et al. 2018; Nyadanu et al. 2022; Richards et al. 2022; Savitz & Hu, 2021; Wang et al. 2019; Yang et al. 2022). High temperature was also defined with various cutpoints, the most common definition being maximum temperature at or above the 95th percentile (Bonell et al. 2025).

Few studies have examined the effects of high ambient temperature on stillbirth outcomes. Most published studies have been conducted in climates concerning tropical, sub-tropical and temperate regions (Bonell et al. 2023). Five previous studies have been conducted in Australia, in subtropical Brisbane, Queensland (Li et al. 2018; Strand et al. 2011; Wang et al. 2019) and in the tropical and mediterranean climate of Western Australia (Nyadanu et al. 2022, 2024). We have identified no published studies that explore the effects of high ambient temperature on stillbirth outcomes in the mediterranean climate (Groves and Di Castri 1991) of SA.

Against the background of increasing temperatures, susceptible population groups may be disproportionately affected by exposure to high temperatures, including pregnant women and their fetus. Understanding this relationship can assist in the development of tailored adaptation and mitigation strategies, targeting the needs of pregnant women across the socio-economic strata.

Variation in stillbirth rates between socially disadvantaged and non-disadvantaged women is apparent (Flenady et al. 2016). It remains unclear from the existing literature how the association between high ambient temperature and stillbirth might vary across the socio-economic spectrum, with only one Australian study considering the effects of high ambient temperature on stillbirth outcomes by social disadvantage (Nyadanu et al. 2022). In high-income countries, women living in social disadvantage have twice the risk of experiencing a stillbirth compared to non-disadvantaged women (Flenady et al. 2016). In addition, the stillbirth rates reported for those living in social disadvantage are likely to be underestimated (Flenady et al. 2016). Thus, the primary aim of our study was to investigate the relationship between maternal exposure to high ambient temperature and stillbirth in SA, overall and stratified by social disadvantage.

## Materials and methods

### Study design

We constructed a retrospective birth cohort of all births in SA between 1 January 2000 and 31 December 2021, linked to publicly available maximum temperature data across the same period. Our study is reported according to the Strengthening the Reporting of Observational Studies in Epidemiology (STROBE) guidelines for cohort studies (https://www.strobe-statement.org/). The study was approved by the South Australian Department for Health and Wellbeing, Human Research Ethics Committee (HRE000109), and endorsed by Adelaide University.

### Data sources

#### Birth cohort

Data were obtained from the South Australian Perinatal Statistics Collection, managed by the Pregnancy Outcome Unit, Preventive Health SA. This collection includes data on live births, stillbirths, and pregnancy terminations compiled from daily birth notifications from hospital midwives, privately practising midwives attending homebirths, and neonatal nurses with data drawn from the Supplementary Birth Record data collection form or its electronic equivalent (Perinatal Statistics Collection & Wellbeing SA, 2023). Birth information including sex (male and female), birthweight, gestational age, birth setting (hospital, birth centre, home and other (including born before arrival)) and hospital category (metropolitan teaching, metropolitan private, country and home), were obtained from the collection. In addition to birth information, maternal characteristics including maternal country of birth (aggregated to include Australia, Asia, Americas, Africa and Europe), occupation, plurality (single and multiple) and age, were also obtained from the collection.

#### Temperature data

Publicly available temperature data were obtained from Scientific Information for Land Owners (SILO) website (https://www.longpaddock.qld.gov.au/silo/gridded-data/). Gridded daily maximum temperature were used in this study. The SILO grid spans the region 112°E to 154°E, 10°S to 44°S, with 0.05° x 0.05° spatial resolution (approximately 5 km x 5 km) (SILO, 2021). We used ArcGIS software (version 10.8.1) to convert daily maximum temperature into extractable data for each day in the study period, from 1 January 2000 to 31 December 2021, at the Statistical Areas Level 3 (SA3).

#### Geographic and socio-economic data

Mother’s place of residence was reported and aggregated by the South Australian Perinatal Statistics Collection to 2016 SA3 using the definition outlined by the Australian Bureau of Statistics (ABS). SA3s are geographical areas which comprise regional towns and cities, or clusters of related suburbs within major urban areas and have populations between 30,000 and 130,000 people (ABS, 2021). We extracted daily maximum temperature for each of the 28 SA3 regions in SA throughout the study period.

We also linked SA3 data to the area-level 2016 Index of Relative Socio-Economic Disadvantage (IRSD) deciles obtained from the ABS. This index assigns a score to each geographic area based on population attributes, such as proportion of the area’s population with low income, low educational attainment, and high unemployment, with the information drawn from the five-yearly Census of Population and Housing (ABS, 2023). IRSD deciles indicate areas of most disadvantage (1) to least disadvantage (10). We calculated the proportion of pregnant women living in social disadvantage in each SA3, using the three lowest SEIFA-IRSD deciles, representing areas of the greatest disadvantage. Based on these proportions, we categorised pregnant women as residing in low, medium or high social disadvantage, where SA3 regions with the largest proportion of disadvantage (i.e. regions with the greatest number of people in the lowest SEIFA-IRSD deciles) defined as high.

### Outcome variable

The outcome of interest in this study was stillbirth, defined as “the birth of a fetus at or after 20 weeks gestation or with a birthweight of 400 g or more, with no signs of life at birth” (Preventive Health 2024).

### Exposure period

An acute exposure period was investigated for the 7 days prior to delivery. We considered the lagged effects of daily maximum temperature on stillbirth from lag day 0 (i.e. daily maximum temperature on delivery) through to lag day 7 (daily maximum temperature 7 days before delivery).

We considered maternal exposure to daily maximum temperature in two ways. According to the existing literature high ambient temperature is most often defined at or above the 95th percentile (Bonell et al. 2025). Therefore, using temperature data from each day in the study period (1 January 2000 to 31 December 2021), we defined high ambient temperature as a binary variable using the cut-point of the 95th percentile (36.1 °C). We also considered temperature as a continuous variable and explored non-linear effects using natural cubic splines with knots at the 10th (14.4 °C), 50th (21.2 °C) and 90th (32.9 °C) percentiles of daily maximum temperature. Percentiles were calculated based on daily maximum temperature data from the entire study period and did not vary by season.

### Covariates

Potential confounding variables were included in the statistical models, including year of birth (to examine possible time trends), and risk factors for stillbirth identified from the extant literature: plurality, gestational age (preterm (< 37 weeks), term (≥ 37–40 weeks) and overdue (≥ 41 weeks)), maternal age, birth setting and maternal country of birth (aggregated) (Bowman et al. 2024; Gandhi and Page 2024).

### Statistical analysis

Mixed-effects logistic regression models were used to examine the effect of maternal exposure to high ambient temperature on the risk of stillbirth, overall, and stratified by social disadvantage. We estimated odds ratios (OR) and 95% confidence intervals (95% CI) for the contemporaneous effects of maternal exposure to high ambient temperature at birth. We separately fit models for exposure to high ambient temperature for each individual day in the week preceding delivery (i.e. temperature lag effects for days 1 through 7 before delivery). We included the potential confounding variables as fixed effects and SA3 as a random effect in our models. Maternal exposure to daily maximum temperature was considered using two distinct methods: first, as a binary variable at or above the 95th percentile (36.1 °C) for each day in the exposure period, and second, as a continuous variable. Regardless of whether temperature was considered as a binary or continuous variable, separate models were fit for each lag day (lag of 0–7 days). To examine how any observed relationship might differ according to social disadvantage, we considered pregnant women living in low, medium or high disadvantage in SA as adjustments in our statistical models.

All statistical analyses were carried out in accordance with a pre-specified statistical analysis plan and performed using Stata software (Release 18. College Station, TX: StataCorp LLC, 2023).

### Sensitivity analysis

We performed three separate sensitivity analyses. First, we created a new outcome variable with the addition of 24 stillbirth and 3,139 livebirth records with indeterminate gestational age to the primary analysis dataset. Second, we analysed a composite outcome variable of stillbirth and neonatal death incorporating the 928 neonatal deaths along with the 2,776 stillbirths. Finally, including the records with indeterminate gestational age to the primary analysis dataset, we analysed a composite outcome variable of stillbirth and neonatal death incorporating the 2,800 stillbirths and 939 neonatal deaths, and 417,570 livebirth records. We fit the same statistical models as outlined previously, with the exclusion of gestational age as a covariate, to each of these datasets, to see if findings differed from our primary analyses.

## Results

**Fig. 1:**
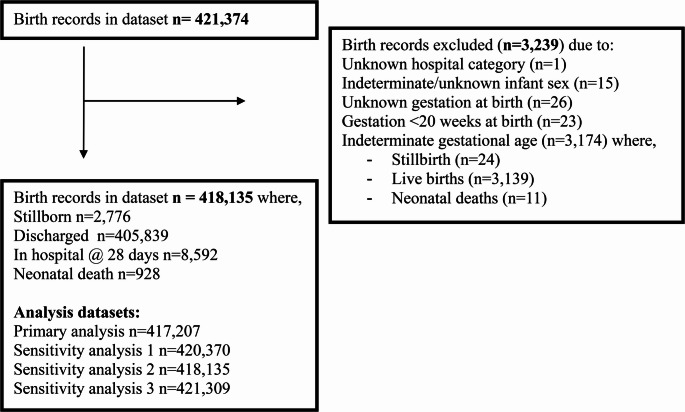
Flow chart of the inclusion and exclusion of birth records

A total of 421,374 birth records, including 2,824 (0.7%) stillbirths (6.7 per 1,000 births), were reported in SA from 1 January 2000 to 31 December 2021. As shown in Fig. 1, our primary analysis dataset comprised 417,207 records, including 2,776 (0.7%) stillbirths and 414,431 live births. Neonatal deaths and records with indeterminate gestational ages were not included in our primary analysis.

Descriptive statistics of daily maximum temperature in SA during the study period are presented in Table 1. The mean daily maximum temperature in January (summer) was 30.0 °C (SD 6.3 °C), with the mean daily maximum temperature 15.1 °C (SD 2.4 °C) in July (winter) across these 21 years.

**Table 1:** Summary of daily maximum temperature (°C) in South Australia by months 2000 to 2021

Month	Mean °C	Standard deviation	Median °C	75^th^ %ile^b^ °C	95^th^ %ile^b^ °C	99^th^ %ile^b^ °C
January	30.0	6.3	29.1	34.5	41.5	44.2
February	29.0	5.8	28.2	33.6	39.1	42.1
March	26.6	5.3	25.6	30.3	36.3	39.5
April	23.0	4.6	22.2	26.0	31.6	35.0
May	18.6	3.3	18.1	20.4	25.0	28.5
June	15.7	2.3	15.5	16.9	19.5	22.3
July	15.1	2.4	14.9	16.3	19.3	21.7
August	16.2	3.2	15.5	17.7	22.4	25.5
September	19.0	4.3	17.9	21.8	27.5	30.7
October	22.3	5.5	21.2	26.0	32.7	36.4
November	25.7	6.2	24.7	30.4	36.7	39.9
December	27.7	6.3	26.4	32.0	39.5	42.7

^a^ The seasons in South Australia are defined as summer months: December, January and February; autumn months: March, April and May; winter months: June, July and August; spring months: September, October and November

^b^ %ile = percentile

Table 2 presents the demographic characteristics of the mothers and babies in our analysis dataset. The mean gestational age for a stillbirth was 27.1 (SD 7.1) weeks, with the majority (82.7%) of stillbirths delivered preterm (< 37 weeks). Slightly over half (51.9%) of stillborn babies were male. Single pregnancies accounted for 92.4% of stillbirths. Most mothers who experienced a stillbirth were born in Australia (77.6%). Mothers aged ≤ 19 years and ≥ 40 years had the highest proportion of stillbirths; 0.9% and 1.0% of all births in those age bands, respectively.

**Table 2:** Demographic characteristics of mothers and babies in the South Australian Perinatal Statistics Collection, 2000 to 2021. Shown are n and row %

	Outcome		
	Stillbirths *n*=2,776 (0.7%)	Livebirths *n*=414,431	Total *n*=417,207
Babies			
*Sex*			
Male	1,442 (0.67%)	212,831	214,273
Female	1,334 (0.66%)	201,600	202,934
*Birthweight*			
Low birthweight <2500 g	2,265 (7.82%)	26,702	28,967
≥2500 g	511 (0.13%)	387,729	388,240
*Gestational age*			
Term (≥37 weeks)	479 (0.13%)	380,735	381,214
Preterm (<37 weeks)	2,297 (6.38%)	33,696	35,993
*Birth setting*			
Hospital	2,736 (0.70%)	386,981	389,717
Birth centre	5 (0.02%)	23,724	23,729
Home	14 (0.71%)	1,964	1,978
Other (including born before arrival)	21 (1.18%)	1,762	1,783
*Hospital category*			
Metropolitan teaching (includes non-maternity facilities)	2,262 (0.96%)	232,424	234,686
Metropolitan private	248 (0.25%)	98,572	98,820
Country (includes non-maternity facilities)	253 (0.31%)	81,495	81,748
Home	13 (0.67%)	1,940	1,953
**Mother**			
*Maternal age*			
≤19	138 (0.91%)	15,107	15,245
20-24	431 (0.74%)	57,581	58,012
25-29	718 (0.60%)	118,473	119,191
30-34	827 (0.59%)	138,588	139,415
35-39	513 (0.73%)	69,898	70,411
≥40	149 (1.00%)	14,784	14,933
*Plurality*			
Single	2,566 (0.63%)	401,975	404,541
Multiples	210 (1.66%)	12,456	12,666
*Maternal country of birth (aggregated)*			
Australia	2,154 (0.65%)	329,660	331,814
Asia	345 (0.74%)	46,498	46,843
Americas	24 (0.59%)	4,031	4,055
Africa	117 (0.89%)	12,976	13,093
Europe	136 (0.64%)	21,266	21,402
*Maternal occupation*			
Managers, administrators & clerks	355 (0.44%)	79,669	80,024
Professionals & para-professionals	488 (0.51%)	94,308	94,796
Tradespersons, labourers, plant & machinery operators & drivers	170 (0.59%)	28,507	28,677
Salespersons & personal service workers	367 (0.58%)	62,798	63,165
Student	109 (0.72%)	15,097	15,206
Home duties	726 (0.73%)	98,458	99,184
Invalid pensioner/pensioner & unemployed	186 (0.98%)	18,788	18,974
Other/unknown	375 (2.18%)	16,806	17,181

**Fig. 2:**
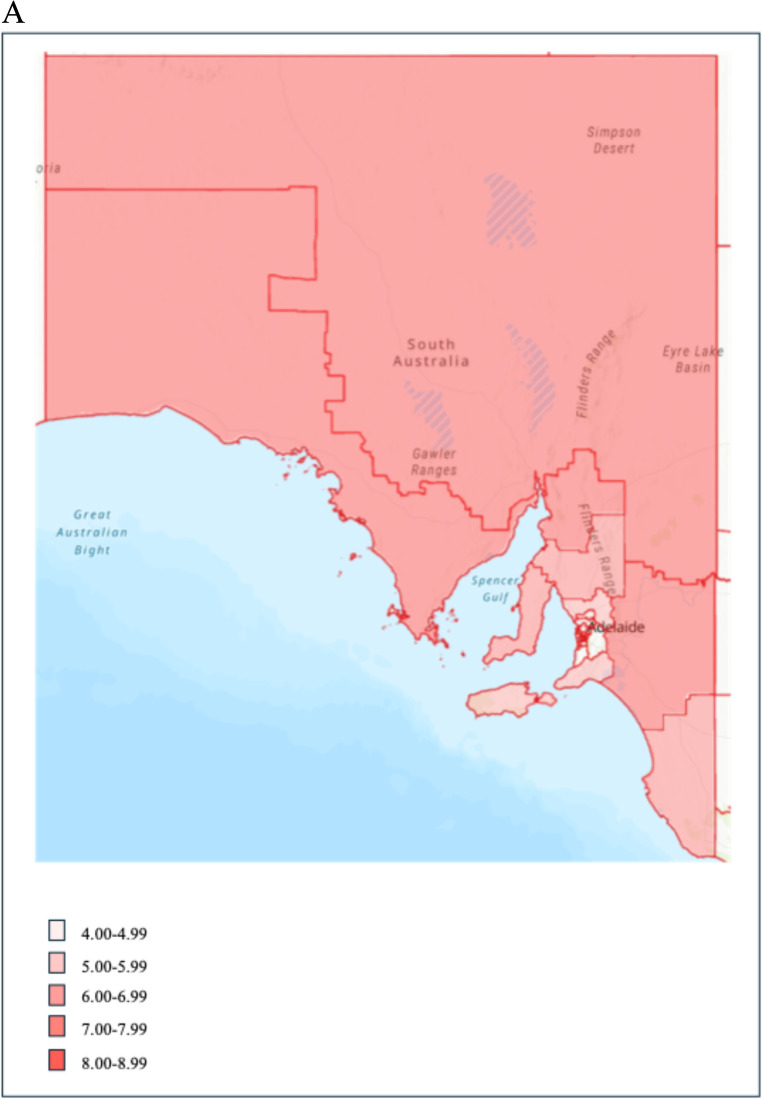

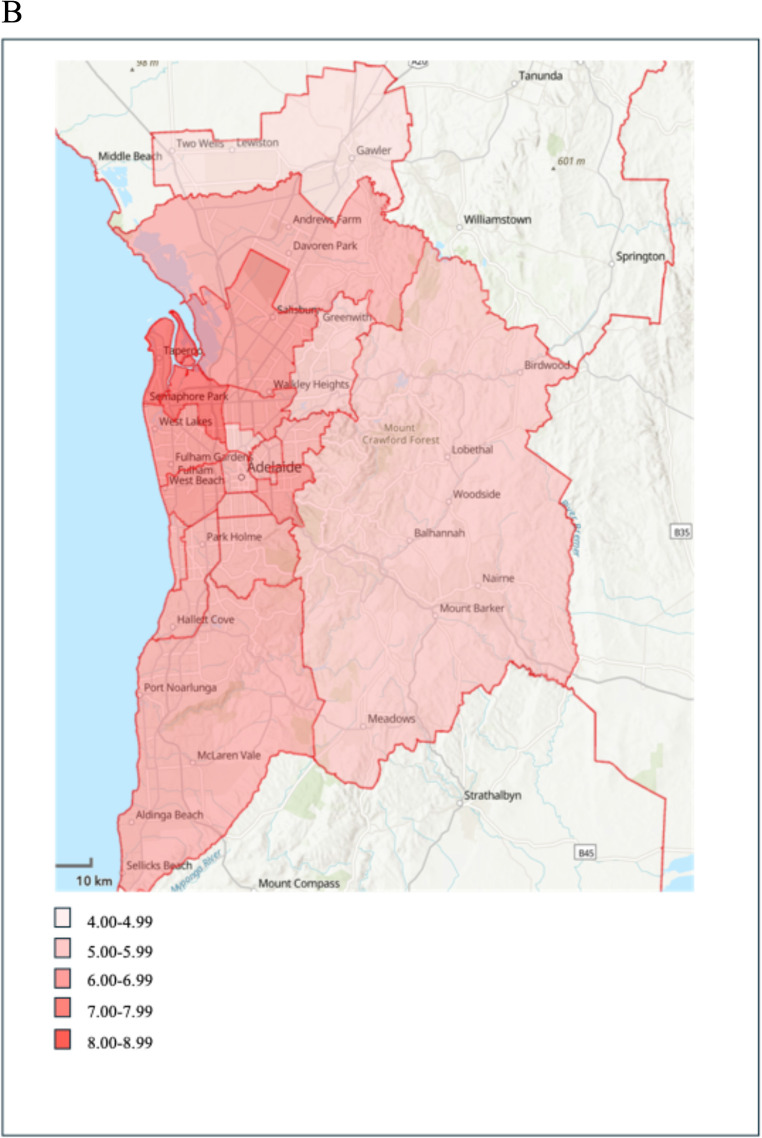
**a** Distribution of stillbirths per 1000 births across regional South Australia, 2000 to 2021. **b** Distribution of stillbirths per 1000 births across metropolitan Adelaide, 2000 to 2021

Figure 2a and b show the distribution of stillbirths per 1000 births according to SA3 regions. The highest rates of stillbirths were observed for mothers living in the metropolitan area of Port Adelaide - West (8.5 per 1000 births), and regional areas of Mid North (7.8 per 1000 births) and Murray and Mallee (7.5 per 1000 births) (Supplementary material Table 1).

**Fig. 3:**
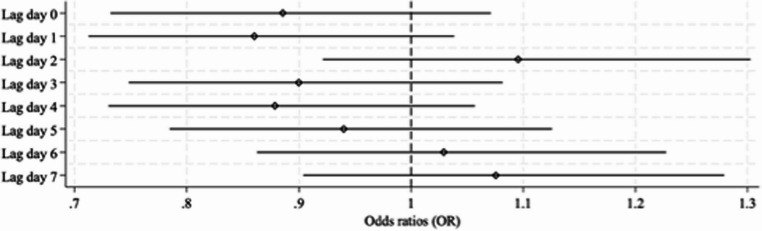
Adjusted odds ratios and 95% confidence intervals on individual lag days for the association between maternal exposure to high ambient temperature and stillbirth using binary temperature at the 95th percentile. All models adjusted for potential confounders including year of birth, plurality, SEIFA IRSD 2016, gestational age, maternal age, birth setting and maternal country of birth. Note:  denotes the odds ratio

Mixed-effects logistic regression models, adjusted for year of birth, plurality, social disadvantage, gestational age, maternal age, birth setting and maternal country of birth, for lag day 0 showed little evidence of an association between high daily maximum temperature ≥ 95th percentile and stillbirth, with an odds ratio slightly less than one (aOR: 0.88, 95%CI: 0.73–1.06, *p* = 0.19) (Fig. 3).

When the week prior to delivery was examined (lag days 1–7), maximum temperature ≥ 95th percentile showed little effect on the odds of stillbirth. The relationship between high ambient temperature and stillbirth was ambiguous with increased risks on lag day 2 (aOR: 1.10, 95%CI: 0.93–1.30, *p* = 0.26), lag day 6 (aOR: 1.06, 95%CI: 0.89–1.25, *p* = 0.53) and lag day 7 (aOR: 1.06, 95%CI: 0.89–1.26, *p* = 0.50). Decreased risks were observed on lag day 0 (aOR: 0.89, 95%CI: 0.73–1.07, *p* = 0.21), lag day 1 (aOR: 0.86, 95%CI: 0.71–1.04, *p* = 0.12), lag day 3 (aOR: 0.90, 95%CI: 0.75–1.08, *p* = 0.26), lag day 4 (aOR: 0.88, 95%CI: 0.73–1.06, *p* = 0.21) and lag day 5 (aOR: 0.94, 95%CI: 0.78–1.13, *p* = 0.21). However, the confidence intervals were wide and included 1, consistent with a null effect (Fig. 3).

The contemporaneous and lagged effects of daily maximum temperature considered as a continuous variable also appeared to show minimal effect on the odds of stillbirth (lag days 1–7; results not shown).

Considering social disadvantage into the statistical models made negligible difference to the relationship between high temperature and stillbirth in the week prior to delivery. Maximum temperature ≥ 95th percentile and high levels of social disadvantage marginally increased the risk of stillbirth for each day in the week prior to delivery compared to low levels of disadvantage (lag day 0 (aOR: 1.03, 95%CI: 0.92–1.17, *p* = 0.59), lag day 2 (aOR: 1.03, 95%CI: 0.91–1.16, *p* = 0.64) and lag day 7 (aOR: 1.03, 95%CI: 0.91–1.16, *p* = 0.64)).

### Sensitivity analysis

When the outcome variable was updated to include neonatal deaths as well as the addition of records with indeterminate gestational age, there were negligible differences in the results from these sensitivity analyses compared to the primary analyses (presented in Supplementary material, Tables 3, 4 and 5).

## Discussion

We examined the effect of maternal exposure to high ambient temperature on the risk of stillbirth, overall and by social disadvantage in the week prior to delivery. Maternal exposure to high ambient temperature ≥ 95th percentile (36.1 °C) showed an ambiguous risk of stillbirth with increases on lag days 2, 6 and 7, and decreases on lag days 0, 1, 3, 4 and 5; as the confidence intervals included 1 there is limited evidence of an effect. Taking social disadvantage into account in the statistical models made little difference to this finding. Maximum temperature ≥ 95th percentile and high levels of social disadvantage compared to low levels of disadvantage marginally increased the risk of stillbirth.

Our findings broadly align with those reported in other studies. In Florida, USA, no overall association between high ambient temperature in the week prior to delivery and stillbirth was demonstrated (Savitz and Hu 2021). In that study, there was evidence of a weak association for temperature above the 90th percentile (31.9 °C), which was more pronounced among the most socio-economically deprived communities in the week prior to delivery (Savitz and Hu 2021). In contrast, we observed minimal effect when considering the association between high temperature and stillbirth by social disadvantage in the week prior to delivery. Potential reasoning for the differences in findings could include suboptimal antenatal care, which contributes to a large proportion of stillbirths (Bowman et al. 2024). In Australia, residents and citizens have access to publicly funded universal healthcare (Angeles et al. 2023), regardless of socio-economic status. Universal healthcare is not available in the USA and suboptimal antenatal care disproportionately affects those living in social disadvantage (Flenady and Ellwood 2020; U.S. Department of Health and Human Services., 2021). In contrast, increased risk of stillbirth was identified for elevated maximum temperature (> 28 °C) in a Canadian study, for up to 6 days prior to birth (Auger et al. 2017). Similarly, the risk of stillbirth increased for each individual day, and cumulatively, in the week prior to delivery in Utah, USA for high temperature > 90th percentile (20 °C) (Kanner et al. 2020). Comparable to our study, both Auger et al., and Kanner et al., observed that the strongest association between high temperature and stillbirth occurred on lag day 2 (Auger et al. 2017; Kanner et al. 2020), which is consistent with the evidence that intrauterine fetal death often occurs approximately 48 h prior to delivery (Gardosi et al. 1998).

There are several plausible mechanisms that have linked maternal exposure to high ambient temperature and stillbirth. Fetal core temperature is around 0.5 °C higher than maternal core temperature and is dependent on maternal temperature, placental blood flow and fetal metabolism (Mehta et al. 2023; Samuels et al. 2022). If maternal core temperature increases, then it is likely that heat is transferred to the fetus (Mehta et al. 2023). Any physiological or systemic effects which interfere with the maternal-fetal heat exchange are likely to impact fetal development and growth, thereby increasing the risk of stillbirth (Gardosi et al. 2013; Mehta et al. 2023). The placenta is dependent on cardiac output for perfusion, and it has been hypothesised that during extremely high ambient temperature exposure, placental perfusion may decrease to allow increased blood flow to the skin, hence increasing a mother’s ability to sweat effectively (Basu et al. 2016; Samuels et al. 2022). Persistent reduction in placental blood flow can result in fetal growth restriction (Samuels et al. 2022), which is a known risk factor for stillbirth (Bonell et al. 2023; Gardosi et al. 2013). Small changes in ambient temperature may impact the thermoregulatory capabilities of the mother, and therefore cause adverse birth outcomes, including stillbirth (Kanner et al. 2020). The thermoregulatory capabilities may be exacerbated for mothers who are already at higher risk at increased ambient temperature, including those living in social disadvantage (Nyadanu et al. 2022).

Our findings must be interpreted considering several strengths and limitations. First, our birth cohort spans the two most recent decades. Therefore, we were able to examine contemporary time trends between high ambient temperature and stillbirth. Second, our birth cohort was large, encompassing more than 400,000 births. Third, our whole of population statewide study covered a socio-demographically and geographically diverse birth cohort, whereas previous studies had been conducted in urban centres only. Fourth, we used gridded daily temperature data with a fine spatial resolution. This meant that a mother’s exposure to daily maximum ambient temperature was captured at a finer resolution than if we utilised a mother’s residential proximity to a single weather recording station. Finally, our statistical models included a range of potential confounders that have been previously identified as risk factors for stillbirth (Bowman et al. 2024; Gandhi and Page 2024).

One limitation is that the linked data did not include the exact date of fetal death (prior to delivery as stillborn), but others have estimated that the timing of fetal death is typically within 48 h of delivery (Gardosi et al. 1998). Presently, there are no reliable imaging techniques that produce an accurate time of intrauterine fetal death (Nyadanu et al. 2022). This can lead to potential misclassification of the exposure period. We also cannot rule out effects of unmeasured confounding on our results. We did not have access to data on other identified risk factors for stillbirth including maternal smoking status, parity, gravidity, body mass index and First Nations status (Bowman et al. 2024; Gandhi and Page 2024). Another limitation is that we could not link subsequent pregnancies of mothers in our dataset, and thus the clustering by mother was not accounted for in the analyses. Hence, we could not identify repeated stillbirths that were attributable to genetic causes. Additionally, we considered outdoor ambient temperature only. We did not have information on the mother’s ability to avoid high outdoor temperature or the implementation of adaptation and mitigation strategies including the use of air conditioning. Jurisdictions that experience mediterranean climates, including South Australia, are routinely exposed to high temperatures during summer, often for extended periods. Pregnant women may already be equipped with strategies to avoid extreme heat, thereby reducing their exposure. This may have created misclassification in individual mother’s exposure to high ambient temperature. Prospectively designed studies measuring participants’ time activity patterns including time spent indoors could be a means of providing more nuanced data at an individual level (Mehta et al. 2023). However, our approach of obtaining birth records retrospectively from registries and hospital data collections is routine in this field of research (Mehta et al. 2023). Finally, we linked area level temperature data to individual level outcomes. Therefore, individual mother’s exposure to high ambient temperature is only an estimate. This may have led to errors in the classification of exposure as area-level temperature may not reflect an individual mother’s true exposure to high ambient temperature. However, our approach was similar to other studies in this field of research (Richards et al. 2022; Wang et al. 2019). Additionally, these classification errors are likely to be randomly distributed, and thus not associated with biased estimates of the effects of temperature on risk on stillbirth.

Although beyond the scope of the present study, we recommend future studies consider the potential physiological mechanisms that link maternal exposure to high temperature and stillbirth. Additionally, there may be critical periods of exposure throughout pregnancy that adversely affect the risk of stillbirth. We recommend future studies consider the timing and cumulative effect of high temperature throughout the entire pregnancy period to identify any critical periods of exposure for stillbirth.

## Conclusions

The findings of our study are informative for the expanding field of research on high temperature and adverse birth outcomes, including stillbirth. In the context of our study and the extant literature, we recommend that healthcare providers and policy makers consider education, adaptation and mitigation strategies for pregnant women, recognising the varying needs across the socio-economic strata. As temperatures are predicted to increase for the remainder of the 21st century in Australia and globally, these strategies will be of increasing importance to reduce stillbirths and their devastating impact.

## Electronic Supplementary Material

Below is the link to the electronic supplementary material.

Supplementary Material 1

## Data Availability

The temperature dataset is publicly available from the Scientific Information for Land Owners website (https://www.longpaddock.qld.gov.au/silo/gridded-data/). As per the data use agreement, the dataset used in this study from the South Australian Perinatal Statistics Collection cannot be made available due to sensitive information.
