# Supplementary material for: Maternal exposure to high ambient temperature and risk of stillbirth in South Australia: a statewide cohort study, 2000–2021
Source: Int J Biometeorol. 2026 May 11;70(5):159. doi: 10.1007/s00484-026-03222-4 (PMC13161335; doi:10.1007/s00484-026-03222-4)
Supplement: Supplementary file 1 — Supplementary Material 1 [file 484_2026_3222_MOESM1_ESM.docx]

Maternal exposure to high ambient temperature and risk of stillbirth in South Australia: A statewide cohort study, 2000-2021.

Journal: Int. J. Biometerology

**Author names and affiliations:**

Chelsea Dyer^1^ [chelsea.dyer@adelaide.edu.au](mailto:chelsea.dyer@adelaide.edu.au)

Adriana Milazzo^1^ [adriana.milazzo@adelaide.edu.au](mailto:adriana.milazzo@adelaide.edu.au)

Lynne Giles^1,2^ [lynne.giles@adelaide.edu.au](mailto:lynne.giles@adelaide.edu.au)

^1^School of Public Health, Adelaide University, South Australia, Australia

^2^ Robinson Research Institute, Adelaide University, South Australia, Australia

**Corresponding author:**

Dr Adriana Milazzo

Supplementary material

| Table 1: Distribution of births in South Australia by SA3^a^, 2016 region, 2000 to 2021 | | | | | |
| --- | --- | --- | --- | --- | --- |
| Stillbirth | Stillbirth | Livebirth | Total | Stillbirth per 1000 births | 95% CI |
| **Overall** | 2776 | 414431 | 417207 | 6.7 | (6.4, 6.9) |
| Adelaide City | 21 | 3004 | 3025 | 6.9 | (4.3, 10.6) |
| Adelaide Hills | 97 | 16684 | 16781 | 5.8 | (4.7, 7.0) |
| Barossa | 39 | 7837 | 7876 | 5.0 | (3.5, 6.8) |
| Burnside | 50 | 6835 | 6885 | 7.3 | (5.4, 9.6) |
| Campbelltown (SA) | 80 | 11996 | 12076 | 6.6 | (5.3, 8.2) |
| Charles Sturt | 194 | 26328 | 26522 | 7.3 | (6.3, 8.4) |
| Eyre Peninsula and South West | 113 | 16050 | 16163 | 7.0 | (5.8, 8.4) |
| Fleurieu - Kangaroo Island | 43 | 8190 | 8233 | 5.2 | (3.8, 7.0) |
| Gawler - Two Wells | 39 | 8019 | 8058 | 4.8 | (3.4, 6.6) |
| Holdfast Bay | 43 | 6366 | 6409 | 6.7 | (4.9, 9.0) |
| Limestone Coast | 117 | 16783 | 16900 | 6.9 | (5.7, 8.3) |
| Lower North | 36 | 5241 | 5277 | 6.8 | (4.8, 9.4) |
| Marion | 141 | 22593 | 22734 | 6.2 | (5.2, 7.3) |
| Mid North | 53 | 6766 | 6819 | 7.8 | (5.8, 10.2) |
| Mitcham | 85 | 13675 | 13760 | 6.2 | (4.9, 7.6) |
| Murray and Mallee | 126 | 16758 | 16884 | 7.5 | (6.2, 8.9) |
| Norwood - Payneham - St Peters | 52 | 7926 | 7978 | 6.5 | (4.9, 8.5) |
| Onkaparinga | 267 | 42843 | 43110 | 6.2 | (5.5, 7.0) |
| Outback - North and East | 56 | 7729 | 7785 | 7.2 | (5.4, 9.3) |
| Playford | 186 | 29760 | 29946 | 6.2 | (5.4, 7.2) |
| Port Adelaide - East | 156 | 20868 | 21024 | 7.4 | (6.3, 8.7) |
| Port Adelaide - West | 127 | 14841 | 14968 | 8.5 | (7.1, 10.1) |
| Prospect - Walkerville | 31 | 6171 | 6202 | 5.0 | (3.4, 7.1) |
| Salisbury | 298 | 39921 | 40219 | 7.4 | (6.6, 8.3) |
| Tea Tree Gully | 133 | 23524 | 23657 | 5.6 | (4.7, 6.7) |
| Unley | 57 | 8641 | 8698 | 6.6 | (5.0, 8.5) |
| West Torrens | 103 | 14286 | 14389 | 7.2 | (5.8, 8.7) |
| Yorke Peninsula | 33 | 4796 | 4829 | 6.8 | (4.7, 9.6)_ |
| ^a^ Metropolitan SA3 areas include Adelaide City, Adelaide Hills, Burnside, Campbelltown (SA), Charles Sturt, Gawler – Two Wells, Holdfast Bay, Marion, Mitcham, Norwood – Payneham – St Peters, Onkaparinga, Playford, Port Adelaide – East, Port Adelaide – West, Prospect – Walkerville, Salisbury, Tea Tree Gully, Unley and West Torrens. Regional SA3 areas include Barossa, Eyre Peninsula and South West, Fleurieu – Kangaroo Island, Limestone Coast, Lower North, Mid North, Murray and Mallee, Outback – North and East and Yorke Peninsula. | | | | | |

| Table 2: Association between maternal exposure to high ambient temperature and stillbirth by social disadvantage in the 7 days preceding date of delivery for daily maximum at or above the 95^th^ percentile, 2000 to 2021 | |  |
| --- | --- | --- |
| Stillbirth | Adjusted^a^ OR and 95% CI  Low disadvantage (1.00 (Reference) vs medium disadvantage | Adjusted^a^ OR and 95% CI  Low disadvantage (ref) vs high disadvantage |
| Lag day 0 | 0.97 (0.86, 1.10) | 1.03 (0.92, 1.17) |
| Lag day 1 | 0.97 (0.86, 1.10) | 1.03 (0.92, 1.17) |
| Lag day 2 | 0.97 (0.86, 1.10) | 1.03 (0.91, 1.16) |
| Lag day 3 | 0.97 (0.86, 1.10) | 1.03 (0.92, 1.17) |
| Lag day 4 | 0.97 (0.86, 1.10) | 1.03 (0.92, 1.17) |
| Lag day 5 | 0.97 (0.86, 1.10) | 1.03 (0.91, 1.17) |
| Lag day 6 | 0.97 (0.86, 1.10) | 1.03 (0.91, 1.16) |
| Lag day 7 | 0.97 (0.86, 1.10) | 1.03 (0.91, 1.16) |
| ^a^ Adjusted for potential confounders including year of birth, plurality, SEIFA IRSD 2016, gestational age, maternal age, birth setting and maternal country of birth. | |  |

| Sensitivity analysis 1  Table 3: Association between maternal exposure to high ambient temperature and stillbirth in the 7 days preceding date of delivery for daily maximum at or above the 95^th^ percentile, 2000 to 2021 | | |
| --- | --- | --- |
| Stillbirth | Adjusted^a^  OR | 95% CI |
| Lag day 0 | 0.89 | (0.74, 1.07) |
| Lag day 1 | 0.87 | (0.72, 1.04) |
| Lag day 2 | 1.09 | (0.92, 1.29) |
| Lag day 3 | 0.92 | 0.77, 1.10) |
| Lag day 4 | 0.88 | (0.74, 1.06) |
| Lag day 5 | 0.97 | (0.82, 1.15) |
| Lag day 6 | 1.06 | (0.90, 1.26) |
| Lag day 7 | 1.07 | (0.90, 1.26) |
| ^a^ Adjusted for potential confounders including year of birth, plurality, SEIFA IRSD 2016, maternal age, birth setting and maternal country of birth. | | |

| Sensitivity analysis 2  Table 4: Association between maternal exposure to high ambient temperature and stillbirth in the 7 days preceding date of delivery for daily maximum at or above the 95^th^ percentile, 2000 to 2021 | | |
| --- | --- | --- |
| Stillbirth | Adjusted^a^  OR | 95% CI |
| Lag day 0 | 0.88 | (0.73, 1.06) |
| Lag day 1 | 0.87 | (0.73, 1.05) |
| Lag day 2 | 1.10 | (0.93, 1.30) |
| Lag day 3 | 0.93 | (0.78, 1.11) |
| Lag day 4 | 0.89 | (0.74, 1.06) |
| Lag day 5 | 0.98 | (0.82, 1.16) |
| Lag day 6 | 1.07 | (0.90, 1.26) |
| Lag day 7 | 1.07 | (0.90, 1.26) |
| ^a^ Adjusted for potential confounders including year of birth, plurality, SEIFA IRSD 2016, maternal age, birth setting and maternal country of birth. | | |

| Sensitivity analysis 3  Table 5: Association between maternal exposure to high ambient temperature and stillbirth in the 7 days preceding date of delivery for daily maximum at or above the 95^th^ percentile , 2000 to 2021 | | |
| --- | --- | --- |
| Stillbirth | Adjusted^a^  OR | 95% CI |
| Lag day 0 | 0.89 | (0.74, 1.07) |
| Lag day 1 | 0.87 | (0.72, 1.04) |
| Lag day 2 | 1.09 | (0.92,1.29) |
| Lag day 3 | 0.92 | (0.77, 1.10) |
| Lag day 4 | 0.89 | (0.74, 1.06) |
| Lag day 5 | 0.98 | (0.82, 1.16) |
| Lag day 6 | 1.07 | (0.91, 1.27) |
| Lag day 7 | 1.07 | (0.91, 1.27) |
| ^a^ Adjusted for potential confounders including year of birth, plurality, SEIFA IRSD 2016, maternal age, birth setting and maternal country of birth. | | |
